# Outcomes of septic cirrhosis patients admitted to the intensive care unit: A retrospective cohort study

**DOI:** 10.1097/MD.0000000000027593

**Published:** 2021-11-19

**Authors:** Ralphe Bou Chebl, Hani Tamim, Musharaf Sadat, Saad Qahtani, Tarek Dabbagh, Yaseen M. Arabi

**Affiliations:** a Department of Emergency Medicine, American University of Beirut, Lebanon; b Intensive Care Department, Ministry of National Guard Health Affairs, King Abdullah International Medical Research Center, King Saud Bin Abdulaziz University for Health Sciences, Riyadh, Kingdom of Saudi Arabia.

**Keywords:** cirrhosis, critical care, intensive care, lengths of stay, mortality, sepsis

## Abstract

The aim of this study is to examine the outcome of septic patients with cirrhosis admitted to the intensive care unit (ICU) and predictors of mortality.

Single center, retrospective cohort study.

The study was conducted in Intensive care Department of King Abdulaziz Medical City, Riyadh, Saudi Arabia.

Data was extracted from a prospectively collected ICU database managed by a full time data collector. All patients with an admission diagnosis of sepsis according to the sepsis-3 definition were included from 2002 to 2017. Patients were categorized into 2 groups based on the presence or absence of cirrhosis.

The primary outcome of the study was in-hospital mortality. Secondary outcomes included ICU mortality, ICU and hospital lengths of stay and mechanical ventilation duration.

A total of 7906 patients were admitted to the ICU with sepsis during the study period, of whom 497 (6.29%) patients had cirrhosis. 64.78% of cirrhotic patients died during their hospital stay compared to 31.54% of non-cirrhotic. On multivariate analysis, cirrhosis patients were at greater odds of dying within their hospital stay as compared to non-cirrhosis patients (Odds ratio {OR} 2.53; 95% confidence interval {CI} 2.04 – 3.15) independent of co-morbidities, organ dysfunction or hemodynamic status. Among cirrhosis patients, elevated international normalization ratio (INR) (OR 1.69; 95% CI 1.29-2.23), hemodialysis (OR 3.09; 95% CI 1.76-5.42) and mechanical ventilation (OR 2.61; 95% CI 1.60–4.28) were the independent predictors of mortality.

Septic cirrhosis patients admitted to the intensive care unit have greater odds of dying during their hospital stay. Among septic cirrhosis patients, elevated INR and the need for hemodialysis and mechanical ventilation were associated with increased mortality.

**Article summary**

- Cirrhosis patients are at an increased risk of developing sepsis, sepsis-induced organ failure, and sepsis-related death.
- The aim of this study is to examine the outcome of patients with cirrhosis admitted with sepsis to the intensive care unit (ICU) and predictors of mortality.
- The results of this study have shown that among septic patients admitted to an intensive care unit, a history of cirrhosis was independently associated with an increased hospital mortality.
- This increased mortality is independent of co-morbidities, organ dysfunction or hemodynamic status.
- Given the high mortality associated with cirrhosis septic patients, further studies are needed to look for novel treatment options and strategies targeted for this high-risk patient population.

## 1 Introduction

Cirrhosis patients are at an increased risk of developing sepsis, sepsis-induced organ failure, and sepsis-related death.^[1–3]^ In the U.S. each year, approximately 200,000 patients with cirrhosis are hospitalized of which approximately 10% require ICU care.^[4]^ The true incidences of sepsis and septic shock have not yet been extensively studied in this high-risk population.^[1]^ Research has shown that the increased susceptibility to infection in cirrhosis patients is due to deficiencies in C3 and C4 complement and impairment of macrophage mediated clearance of bacteria.^[5,6]^ Sepsis is associated with poor prognosis in these patients, as reported in-hospital mortality of cirrhosis patients with septic shock reaches 70%.^[1]^ Research done by Sauneuf et al has shown an improvement in sepsis related outcomes amongst cirrhosis patients after the implementation of a new sepsis treatment protocol as they were able to show that the survival of cirrhosis patients increased from 6% between 1997–2004 to 29% in 2005–2010.^[3,7,8]^ However, their study was limited by its small sample size and the literature on this high-risk population is still lacking. The aim of this study is to examine the outcome of patients with cirrhosis admitted with sepsis to the intensive care unit (ICU) and predictors of mortality.

## 2 Methods

This is a single center, retrospective, cohort study conducted in an academic ICU of a large tertiary care center between 2002 and 2017. The ethics approval was obtained from the Institutional board review of National Guard Health affairs (IRBC/0267/20). All patients who were admitted to the adult ICU with sepsis were included in the analysis. The diagnosis of sepsis was constructed based on the variables available in the ICU database according to the sepsis-3 criteria, which was calculated retrospectively, and defined as the presence of an infection with signs of organ dysfunction, which were represented by a Sequential Organ Failure Assessment (SOFA) score of 2 points or greater. Septic shock was defined by a vasopressor requirement to maintain a mean arterial pressure of 65 mm Hg and a serum lactate level greater than 2 mmol/L (>18 mg/dL) in the absence of hypovolemia. When calculating patient's SOFA scores, missing variables were substituted by the mean value for that variable. There were missing data for GCS, PaO_2_/FiO_2_, creatinine and bilirubin in 3.04%, 3.84%, 4.55% and 21% patients respectively. Patients were diagnosed with cirrhosis if they had a liver biopsy proving cirrhosis, or any signs of liver disease with documented portal hypertension, or episodes of past upper GI bleeding attributed to portal hypertension, Prior episodes of hepatic failure or hepatic encephalopathy.^[9]^ Patients were categorized into two groups based on the presence of cirrhosis on admission. Variables that were collected included the patients’ age, sex, acute physiology and chronic health evaluation (APACHE) II score,^[9]^ SOFA score,^[10]^ vital signs at presentation, severe chronic comorbidities as defined by acute physiology and chronic health evaluation (APACHE) II system, history of chronic kidney disease, history of diabetes, Glasgow coma scale (GCS), mechanical ventilation requirement in the first 24 h of admission, the ratio of partial pressure of oxygen to the fraction of inspired oxygen (PaO2 /FiO2), requirement for vasopressors (defined as use of any vasopressor infusion except dopamine <5 μ g/kg/min), admission bilirubin, creatinine, lactate and international normalization ratio (INR). The primary outcome of the study is all-cause in-hospital mortality. Secondary outcomes included ICU mortality in addition to ICU and hospital lengths of stay and mechanical ventilation duration (MVD) among all patients and among survivors only; the latter calculations were made to account for the competing effect of mortality on these variables.

### 2.1 Patient and public involvement

This is a retrospective chart review study where the patients were not involved in the study process. The study results will help guide our future management and identify the higher mortality risk of cirrhosis patient with sepsis.

### 2.2 Statistical analysis

Statistical analysis software (SAS, version 9.0; SAS Institute, Cary, NC, USA) was used to analyze the data. Continuous data are presented as means with standard deviations or medians and interquartile ranges (IQR) as appropriate based on normality distribution. Categorical data were reported as frequencies and percentages. The chi square or ANOVA was used to test significant differences between study groups. To determine the association between cirrhosis and hospital mortality, bivariate and then multivariate logistic regression analyses were performed. The variables entered in the model were selected based on statistical as well as on clinical significance. The variables entered in the model included age, gender, comorbidities (chronic cardiovascular disease, chronic respiratory disease, diabetes, end stage renal disease (ESRD) on hemodialysis (HD), APACHE II, lactic acid, INR and creatinine.

We tested the effect modification as well as the tests of interactions of selected subgroups on the association between cirrhosis and mortality. These subgroups included the following: male versus female, age older than 50 years versus age younger than 50 years, diabetes versus no diabetes, admission diagnosis, mechanical ventilation versus no mechanical ventilation, vasopressor use versus no vasopressor use. Results of logistic regression analysis were reported as odds ratio (OR) and 95% confidence interval (CI). A *P* value <.05 was considered statistically significant.

## 3 Results

### 3.1 Patient characteristics

During the study period, there were 7905 septic patients admitted to the ICU and were included in the study, of whom 498 (6.30%) were known to have cirrhosis. In both groups, the majority of patients were older than 50. Cirrhosis patients had a higher rate of ESRD (18.15% versus 11.48%, <.001). They had however lower chronic respiratory illness (19.19% vs 27.80%, *P* <.001) and chronic cardiac illnesses (15.76% vs 27.37%, *P* < .001). There was no significant difference in diabetes prevalence between both groups (46.39% vs 48.79%, *P* .3). Cirrhosis patients had a higher APACHE II score on admission to the ICU (28.50 (±8.30) vs 23.67 (±27.59) *P* < .001), a higher lactic acid level (5.04 mg/dl (±4.83) vs 2.98 mg/dl (±3.24), *P* < .001), a higher creatinine level (204.3 μmol/l (±150.60) vs 161.8 μmol/l (±157.40), *P* < .001) as well as a higher INR level (2.28 (±1.42) vs 1.54 (±1.03), *P* < .001). (Table 1)

**Table 1:** Baseline characteristics and clinical data among patients with no cirrhosis and cirrhosis who were admitted with sepsis.

Variable	No cirrhosis N = 7408	Cirrhosis N = 498	*P*-value
Demographic data			
Female gender, N (%)	3302 (44.6)	247 (49.6)	.03
Age (year), mean ± SD	61.7 ± 19.8)	62.9 ± 13.6)	.07
Age >50 yr, n (%)	5565 (75.1)	429 (86.1)	<.0001
Chronic comorbidities- n (%)			
Chronic cardiac illness	2023 (27.4)	78 (15.8)	<.0001
Chronic respiratory illness	2054 (27.8)	95 (19.20)	<.0001
Chronic renal disease	848 (11.5)	90 (18.2)	<.0001
Chronic immunosuppression	863 (11.7)	46 (9.3)	.10
Diabetes mellitus	3614 (48.8)	231 (46.4)	.30
APACHE II, mean ± SD	23.7 ± 27.6	28.5 ± 8.3	<.0001
Glasgow coma scale, mean ± SD	11.3 ± 4.0	10.9 ± 4.4	.05
PaO_2_/FiO_2_ ratio, mean ± SD	203.6 ± 116.3	198.8 ± 124.9	.41
Vasopressor use, n (%)	3168 (42.8)	312 (62.7)	<.0001
Mechanical ventilation, n (%)	4565 (61.6)	361 (72.5)	<.0001
Septic shock, n (%)	1721 (23.2)	218 (43.8)	<.0001
Bilirubin (μmol/l), mean ± SD	29.2 ± 63.7	144.8 ± 178.3	<.0001
Creatinine (μmol/l), mean ± SD	161.8 ± 157.4	204.3 ± 150.6	<.0001
Lactic acid (mg/dl), mean ± SD	3.0 ± 3.2	5.0 ± 4.8	<.0001
INR, mean ± SD	1.5 ± 1.0	2.3 ± 1.4	<.0001

APACHE II = Acute Physiology and Chronic Health Evaluation II, INR = International normalized ratio, PaO_2_/FiO_2_ = the ratio of partial pressure of oxygen to the fraction of inspired oxygen.

### 3.2 ICU management

Cirrhosis patients were more likely to get intubated than non-cirrhotic patients (72.49% vs 61.62%, *P* < .001) and to require vasopressors (62.65% vs 42.76%, *P* < .001). There was no statistically significant difference in mechanical ventilation duration or in ICU LOS among survivors. However, hospital LOS among survivors was longer among cirrhotic patients compared to non-cirrhotic patients (27.0 (26.0) vs 23.0 (39.0) *P* < .001).

### 3.3 Patient outcomes

Cirrhosis patients had a higher hospital mortality than non-cirrhotic patients (64.79% vs 31.54%, *P* < .001) as well as a higher ICU mortality (47.47% vs 18.05%, *P* < .001). (Table 2) Multivariable analysis demonstrated that cirrhosis was associated with greater odds of in-hospital mortality (OR 2.53, 95% CI 2.04 to 3.15), as well as ICU mortality (OR 2.41, 95% CI 1.93 to 3.00) while adjusting for other confounders. (Table 2). The most important predictors of hospital mortality in septic cirrhosis patients were found to be mechanical ventilation (OR 2.61; 95% CI 1.60–4.28) and a history of hemodialysis (OR 3.09; 95% CI 1.76–5.42). (Table 3) Finally, there was no difference in the effect modification between cirrhosis and hospital mortality across all subgroups and these results are summarized in Table 4.

**Table 2:** Multivariate analysis for ICU and hospital mortalities and length of stays (LOS) among survivors in patients with no cirrhosis and cirrhosis who were admitted with sepsis.

	No cirrhosis N = 7408	Cirrhosis N = 498	*P*-value	OR (95% CI)	*P*-value
Categorical outcomes					
ICU mortality, N (%)	1322 (18.1)	235 (47.5)	<.0001	2.41 (1.93, 3.00)	<.0001
Hospital mortality, N (%)	2331 (31.5)	322 (64.8)	<.0001	2.53 (2.04, 3.15)	<.0001

Continuous outcomes among all patients				β coefficient (95% CI)	*P*-value
ICU Length of stay, (Median, IQR)	4.4 (9.3)	5.7 (9.8)	.96	−0.56 (−2.68, 1.57)	.61
Hospital length of stay, (Median, IQR)	23.0 (40.0)	21.0 (22.0)	<.0001	−16.60 (−23.75, −9.45)	<.0001
Mechanical ventilation duration, (Median, IQR)	2.0 (8.0)	4.0 (9.0)	.69	−1.76 (−3.46, −0.06)	.04

Continuous outcomes among hospital survivors					
	No cirrhosis N = 5077	Cirrhosis N = 176			
ICU length of stay, (Median, IQR)	3.5 (7.4)	3.5 (8.2)	.98	0.39 (−3.10, 3.87)	.83
Hospital length of stay, (Median, IQR)	23.0 (39.0)	27.0 (26.0)	<.0001	−15.38 (-28.87, −1.88)	.03
Mechanical ventilation duration, (Median, IQR)	1.0 (5.0)	1.0 (5.0)	.32	−1.19 (−3.96, 1.58)	.40

ICU = Intensive care unit. The following variables were included in the model: cirrhosis; hemodialysis; mechanical ventilation duration; INR; vasopressor use; APACHE II; Chronic renal disease; chronic respiratory disease; gender; age, lactate.

**Table 3:** Predictors of hospital mortality among cirrhotic patients admitted with sepsis.

Parameters	Odds ratio (OR)	95% Confidence interval (CI)
Age (per 1-year increase)	1.02	1.00–1.04
Sex	1.24	0.80–1.93
APACHE II (per 1-unit increase)	1.09	1.05–1.14
INR (per 1-unit increase)	1.69	1.29–2.23
Mechanical ventilation	2.61	1.60–4.28
Hemodialysis	3.09	1.76–5.42
Lactic Acid (>2mmol/L)	1.01	0.64–1.60
Vasopressors	1.21	0.76–1.93
Diabetes Mellitus	0.61	0.39–0.96
Chronic cardiovascular	0.65	0.36–1.19
Chronic respiratory illness	1.22	0.69–2.16
Chronic renal illness	0.86	0.43–1.69
Creatinine (per 100 units increments)	0.84	0.71–1.00

APACHE II = Acute Physiology and Chronic Health Evaluation II, INR = International normalized ratio.

**Table 4:** Multivariate subgroup analysis by different baseline characteristics for the association between cirrhosis status and hospital mortality.

	No Cirrhosis N = 2331	Cirrhosis N = 322	OR (95% CI)	*P*-value	*P* value for interaction
Gender					
Male	1342 (32.7)	159 (63.6)	2.27 (1.67–3.08)	<.0001	.73
Female	989 (30.0)	163 (66.0)	2.82 (2.06–3.87)	<.0001	
Age					
<50	382 (20.7)	41 (59.4)	2.04 (1.11–3.74)	.021	.81
>50	1949 (35.1)	281 (65.6)	2.44 (1.92–3.09)	<.0001	
Diabetes					
No	1140 (30.1)	184 (68.9)	2.81 (2.06–3.82)	<.0001	.14
Yes	1191 (33.1)	138 (60.0)	2.04 (1.49–2.80)	<.0001	
Mechanical ventilation					
No	408 (14.4)	52 (37.9)	2.49 (1.70–3.64)	<.0001	.76
Yes	1923 (42.2)	270 (75.0)	2.31 (1.77–3.02)	<.0001	
Vasopressors					
No	921 (21.8)	97 (52.2)	2.60 (1.86–3.64)	<.0001	.5
Yes	1410 (44.6)	225 (72.4)	2.32 (1.74–3.10)	<.0001	
Chronic cardiac disease					
No	1609 (29.9)	279 (66.4)	2.36 (1.84–3.04)	<.0001	.14
Yes	722 (35.8)	43 (55.8)	1.70 (1.01–2.86)	.0432	
Chronic respiratory disease					
No	1737 (32.5)	258 (64.2)	2.13 (1.65–2.73)	<.0001	.33
Yes	594 (28.9)	64 (67.4)	3.05 (1.86–4.99)	<.0001	
Lactic acid					
<2mmol/L	1225 (26.4)	98 (55.7)	2.63 (1.87–3.69)	<.0001	.81
>2mmol/L	1106 (40.2)	224 (69.8)	2.25 (1.68–3.01)	<.0001	
Hemodialysis					
No	1944 (29.7)	258 (63.4)	2.38 (1.86–3.04)	<.0001	.44
Yes	387 (45.9)	64 (71.1)	2.10 (1.25–3.54)	<.0001	

## 4 Discussion

The results of this study have shown that among septic patients admitted to an intensive care unit, a history of cirrhosis was independently associated with an increased hospital mortality. It is important to note that the effect of cirrhosis on hospital mortality was similar across all subgroups. Furthermore, in our cohort of patients 64.8% of cirrhosis patients admitted to the ICU with sepsis died during their hospital stay as compared 31.54% in the non-septic population. Our results are in line with the literature that shows that the overall mortality rate of septic shock remains particularly high in cirrhotic patients, ranging from 60% to 100%.^[1,2,11–13]^ Cirrhosis patients admitted to the hospital with an acute decompensation and organ failure are classified as having acute on chronic liver failure (ACLF) and are at a higher mortality than the non-cirrhosis population.^[4,11]^ Several reasons why they are at an increased risk of mortality form sepsis as compared to the non-cirrhosis patients. First of all, bacterial infections are much more common in patients with cirrhosis than in the general population.^[14]^ Another factor that increases mortality in cirrhosis patients is their predisposition to the development of acute kidney injury and renal failure. Renal failure occurs in 25–50% of patients with cirrhosis with sepsis and worsens the prognosis.^[15–17]^ As evidenced in our cohort, cirrhosis patients had more evidence of kidney disease than non-cirrhotic patients (18.15% vs 11.48%) as well as more patients on dialysis (39.76% vs 17.54%). The increased risk of AKI is multifactorial. It is most likely due to the hypoperfusion of the kidney both from splanchnic vessel vasodilation as well as the hypovolemia that occurs from sepsis which predisposes the patients at risk for pre-renal, acute tubular necrosis (ATN) or hepatorenal syndrome.^[18–20]^ Moreover, in our study, the two most common reasons for admission to the ICU were shock and respiratory. Cirrhosis patients are at a higher risk of developing lung infections because of reduced alveolar macrophage antibacterial activity.^[11,21]^ Furthermore, ACLF patients presenting with altered mental status from hepatic encephalopathy at a higher risk of aspiration pneumonia.^[22]^ As for the hypotension and shock that was seen in 56% of our cirrhosis patient population, it is due to marked splanchnic vasodilatation which could be further compounded by the hypotension that occurs in sepsis and septic shock through the release of inflammatory cytokines^[23]^ This was highlighted in our study, where cirrhosis patients were more likely to be started on vasopressors as opposed to the non-cirrhosis cohort (62.65% vs 42.76%, *P* < .001). In septic shock, one of the main goals is to achieve a mean arterial pressure of 65 mmHg or more.^[24]^ Historically, the treatment of sepsis revolved around aggressive hydration as proposed by the EGDT protocol.^[25]^ However, the current literature suggests that aggressive fluid hydration may worsen outcomes.^[24,26,27]^ Cirrhotic patients, with their low oncotic pressures, are at risk of developing extracellular edema and pulmonary edema with aggressive fluid hydration.^[11]^ It is probably more prudent to start vasopressors early on in the treatment of septic cirrhosis patients to avoid these complications. One interesting finding in our study was that the cirrhosis group had a higher lactate than non-cirrhotic patients (5.04 ± 4.83) versus (2.98 ± 3.24), however lactate was not found to be a predictor of mortality on septic cirrhosis patients. Laboratory markers of shock should be interpreted with caution in cirrhosis patients; lactate levels are usually higher in patients with cirrhosis as compared to patients without cirrhosis due to decreased lactate clearance by the liver.^[28]^ Therefore, lactate clearance and trends may be more informative than absolute values. Additionally, when we looked at lengths of stays, we noticed that the ICU LOS was similar between both cohorts but the hospital LOS was significantly shorter for the cirrhosis group. One possible explanation is that cirrhosis patients present with low blood pressures and elevated lactate levels and warrant ICU admission for monitoring. However, this can be due to the pathophysiology of cirrhosis and not necessarily due to sepsis. Therefore, the patients that did survive to discharge might have responded quickly to antibiotics and improved clinically which could explain the shorter LOS.

Finally, in our study, the predictors of ICU and hospital mortality amongst septic cirrhosis patients were an elevated INR, need for mechanical ventilation and need for renal replacement. This is similar to reports in the literature that looked at predictors of mortality amongst cirrhosis patients. The INR is a marker of liver activity and has been incorporated in many prognostic scoring scores such as the MELD-Na, and the Child-Pugh scores.^[8]^ Furthermore, according to a study by Sauneuf et al determinants of hospital mortality in cirrhosis patients were the stage of the liver disease as determined by the Child-Pugh score, and the extent of organ failures such as the need for renal replacement therapy.^[3]^ Finally, in a study by Rabe et al, Patients with cirrhosis who required mechanical ventilation had mortality and as high as 100%.^[29]^ The overall outcome of sepsis has improved over the recent years, owing to rapid recognition and early antibiotics.^[24,30]^ Cirrhotic patients are usually left out of interventional trials in sepsis and further studies should be done to evaluate this high risk population.

## 5 Limitations

This was a retrospective study and as such, the authors are aware of the inherent limitations of such a type of study. However, we believe that this was overcome by the large sample size of the study. Furthermore, this study is single centered, from a tertiary care center that is a referral for the region and deals with patients with high acuity which would explain the high mortality rate seen in both cohorts. In our study, details about the severity of cirrhosis and its associated complications such as MELD scores or the child-pugh are not available. These scores have been shown to be a predictor of mortality in different studies.^[31]^

## 6 Conclusion

Septic patients admitted to the intensive care unit with a known history of cirrhosis have an increased ICU and hospital mortality. This increased mortality is independent of co-morbidities, organ dysfunction or hemodynamic status. Given the high mortality associated with cirrhosis septic patients, further studies are needed to look for novel treatment options and strategies targeted for this high-risk patient population.

## Author contributions

**Conceptualization:** Ralph Bou Chebl, Hani Tamim, Musharaf Sadat, Tarek Dabbagh, Yaseen Arabi.

**Data curation:** Ralph Bou Chebl, Hani Tamim, Musharaf Sadat, Saad Qahtani, Tarek Dabbagh, Yaseen Arabi.

**Formal analysis:** Ralph Bou Chebl, Hani Tamim, Musharaf Sadat, Saad Qahtani, Tarek Dabbagh, Yaseen Arabi.

**Funding acquisition:** Ralph Bou Chebl, Hani Tamim, Musharaf Sadat, Saad Qahtani, Tarek Dabbagh, Yaseen Arabi.

**Investigation:** Ralph Bou Chebl, Hani Tamim, Musharaf Sadat, Saad Qahtani, Tarek Dabbagh, Yaseen Arabi.

**Methodology:** Ralph Bou Chebl, Hani Tamim, Musharaf Sadat, Saad Qahtani, Tarek Dabbagh, Yaseen Arabi.

**Project administration:** Ralph Bou Chebl, Hani Tamim, Musharaf Sadat, Saad Qahtani, Tarek Dabbagh, Yaseen Arabi.

**Resources:** Ralph Bou Chebl, Hani Tamim, Musharaf Sadat, Saad Qahtani, Tarek Dabbagh, Yaseen Arabi.

**Software:** Ralph Bou Chebl, Hani Tamim, Musharaf Sadat, Saad Qahtani, Tarek Dabbagh, Yaseen Arabi.

**Supervision:** Ralph Bou Chebl, Hani Tamim, Musharaf Sadat, Saad Qahtani, Tarek Dabbagh, Yaseen Arabi.

**Validation:** Ralph Bou Chebl, Hani Tamim, Musharaf Sadat, Saad Qahtani, Tarek Dabbagh, Yaseen Arabi.

**Visualization:** Ralph Bou Chebl, Hani Tamim, Musharaf Sadat, Saad Qahtani, Tarek Dabbagh, Yaseen Arabi.

**Writing – original draft:** Ralph Bou Chebl, Hani Tamim, Musharaf Sadat, Saad Qahtani, Tarek Dabbagh, Yaseen Arabi.

**Writing – review & editing:** Ralph Bou Chebl, Hani Tamim, Musharaf Sadat, Saad Qahtani, Tarek Dabbagh, Yaseen Arabi.
